# Lutein and zeaxanthin supplementation reduces H_2_O_2_-induced oxidative damage in human lens epithelial cells

**Published:** 2011-12-07

**Authors:** Shasha Gao, Tingyu Qin, Zhenzhen Liu, Maria Andrea Caceres, Carlos F. Ronchi, C-Y. Oliver Chen, Kyung-jin Yeum, Allen Taylor, Jeffery B. Blumberg, Yizhi Liu, Fu Shang

**Affiliations:** 1 State Key Laboratory of Ophthalmology, Zhongshan Ophthalmic Center, Sun Yat-sen University, Guangzhou, China; 2 Jean Mayer USDA Human Nutrition Research Center on Aging, Tufts University, Boston, MA

## Abstract

**Purpose:**

Epidemiological studies suggest that dietary intake of lutein and zeaxanthin is inversely related to the risk for senile cataract. The objectives of this work were to investigate the mechanisms by which these nutrients provide anti-cataract effects. We evaluated their modulation of oxidative damage in human lens epithelial cells (HLEC) and their interaction with intracellular glutathione (GSH).

**Methods:**

Subconfluent HLEC were pre-incubated with or without 5 µM lutein, zeaxanthin, or α-tocopherol for 48 h and then exposed to 100 µM H_2_O_2_ for 1 h. Levels of protein carbonyls in the cells were measured by western-blotting analysis following reaction with 2,4-dinitrophenylhydrazine (DNPH). Levels of malondialdehyde (MDA), reduced glutathione (GSH) and oxidized glutathione (GSSG) were measured by an HPLC system. DNA damage was assessed using comet assays. Cell viability was determined by 3-(4,5-dimethylthiazol-2-yl)-5-(3-carboxymethoxyphenyl)-2-(4-sulfophenyl)-2H-tetrazolium (MTS) assay.

**Results:**

In the absence of H_2_O_2_, HLEC had very low levels of protein carbonyl and MDA. Supplementation with lutein, zeaxanthin, or α-tocopherol to the unstressed HLEC had no detectable effects on levels of oxidized proteins and lipid in the cells. Exposure of HLEC to H_2_O_2_ significantly increased levels of oxidized proteins, lipid peroxidation, and DNA damage. Pre-incubation with lutein, zeaxanthin, or α-tocopherol dramatically reduced the levels of H_2_O_2_ -induced protein carbonyl, MDA, and DNA damage in HLEC. The protective effects of lutein, zeaxanthin, and α-tocopherol against protein oxidation, lipid peroxidation, and DNA damage were comparable. Supplementation with lutein, zeaxanthin, or α-tocopherol increased GSH levels and GSH:GSSG ratio, particularly in response to oxidative stress. Depletion of GSH resulted in significant increase in susceptibility to H_2_O_2_-induced cell death. Supplementation with α-tocopherol, but not lutein or zeaxanthin, can partially restore the resistance of GSH-depleted cells to H_2_O_2_.

**Conclusions:**

These data indicate that lutein or zeaxanthin supplementation protects lens protein, lipid, and DNA from oxidative damage and improves intracellular redox status upon oxidative stress. The protective effects are comparable to that of α-tocopherol, except that lutein and zeaxanthin cannot compensate for GSH depletion. The data imply that sufficient intake of lutein and zeaxanthin may reduce the risk for senile cataract via protecting the lens from oxidative damage.

## Introduction

Cataract is the leading cause of blindness and visual impairment in the world [1]. The incidence of cataract continues to increase with the growing elderly population. Cataract surgery is still the only effective treatment for this disease [2]. Strategies to reduce the risk and/or to delay the development of senile cataract would ameliorate visual impairments and reduce the cost associated with this disease. Improvement of dietary intake of micronutrients might be one of the strategies for reducing the risk for senile cataract. Lutein and zeaxanthin are among the micronutrients that have been reported to be associated with reduced risk for senile cataract. Epidemiologic studies indicate that high dietary intake or blood levels of lutein or zeaxanthin are associated with decreased risk of cataract [3-9]. Lutein and zeaxanthin are isomers with identical chemical formulas-C_40_H_56_O_2_. Like other carotenoids, all of the lutein and zeaxanthin in the body are obtained from diets or supplements. Lutein and zeaxanthin are found in a broad spectrum of foods, such as yellow corn, egg yolk, parsley, spinach, and other fruits or green leafy vegetables. Lutein and zeaxanthin are the only carotenoids detected in the lens [10]. We have found that the lutein and zeaxanthin in the lens are not evenly distributed. The concentrations of lutein and zeaxanthin decrease from the epithelium to the nucleus [11]. The role of lutein and zeaxanthin in the lens remains unknown and the molecular mechanisms by which increased lutein or zeaxanthin intake may reduce the risk for cataract remain to be elucidated.

Oxidative stress is one of the major risk factor for senile cataract, particularly nuclear cataract [12,13]. Exposure to oxidative stress results in lens opacification both in experimental animal models [14,15] and in cultured lens systems [16-18]. Elevated levels of oxidative stress marker were also observed in blood of cataract patients [19]. An increase in levels of antioxidants in the lens would prevent or ameliorate oxidative damage and reduce the risk for cataract [18]. Lutein and zeaxanthin are lipid soluble antioxidants and it is proposed that the benefit of increased lutein and zeaxanthin intake may be related to their antioxidant properties [20,21].

H_2_O_2_ is one of the physiologically relevant oxidants in the lens and in the aqueous humor [22]. Levels of H_2_O_2_ in the aqueous humor of individuals with cataracts are higher than those in the aqueous humor of normal individuals [23,24]. Exposure of the lens to physiologically relevant levels of H_2_O_2_ in vitro results in protein oxidation, lipid peroxidation, and DNA damage as well as lens opacification [25]. We used cultured lens epithelial cells as a model system to study the effects of supplementation of lutein or zeaxanthin on protein oxidation, lipid peroxidation, DNA damage, cellular redox status, and cell viability upon exposure to H_2_O_2._ We used α-tocopherol as a positive control in this study since it is the most studied lipid-soluble antioxidant and we previously demonstrated that supplementation of α-tocopherol to rabbit lens epithelial cells can improve cellular redox status and restore the resistance of GSH-depleted cells to H_2_O_2_ [26]. Results of this study show that supplementation of lutein or zeaxanthin to HLEC reduced protein oxidation, lipid peroxidation and DNA damage upon exposure to H_2_O_2_. The protective effects of lutein or zeaxanthin were comparable to that of same levels of α-tocopherol. These data support the hypothesis that lutein or zeaxanthin may reduce the risk for cataract by protecting lens from oxidative damage.

## Methods

### Materials

D-L(R:S) buthionine sulfoximine (BSO), 2, 4-dinitrophenylhydrazine (DNPH), 30% hydrogen peroxide stock solution, α-tocopherol, DMEM, and anti-DNPH antibody were purchased from Sigma Chemical Co. (St. Louis, MO). Lutein and zeaxanthin were purchased from ChromaDex Inc. (Irvine, CA). The cell Titer 96 aqueous nonradioactive cell proliferation assay (MTS assay) was purchased from Promega (Madison, WI). HRP-conjugated anti-rabbit and anti-mouse secondary antibodies were purchased from Jackson ImmunoResearch Laboratories (West Grove, PA). All organic solvents and glacial acetic acid were purchased from Fisher Co. (Fair Lawn, NJ).

### Cell culture conditions

HLEC (SRA 01/04) were maintained at 37 °C under 5% CO_2_ in DMEM supplemented with 10% heat-inactivated fetal bovine serum,100 units/ml penicillin G and 100 µg/ml streptomycin. To assess the effects on protein oxidation, lipid peroxidation, and DNA damage, cells were seeded in a 60 mm culture dish and subconfluent cells (~90% confluence) were pre-incubated with or without 5 µM of lutein, zeaxanthin or α-tocopherol for 48 h. After removal of the media, cells were rinsed twice with PBS and exposed to 100 µM H_2_O_2_ for 1 h in pyruvate-, phenol red-, and serum-free medium. The cells were then washed twice with cold PBS and collected for different assays.

### Cell viability assay

HLEC were seeded into 96-well plates at ~3000 cells/well and pretreated with 5 µM lutein, zeaxanthin or α-tocopherol together with or without BSO for 48 h. After removal of medium and unincorporated lutein, zeaxanthin, or α-tocopherol, cells were then exposed to the indicated concentrations of H_2_O_2_ in a serum-, pyruvate-, and phenol red-free medium for 1 h. Cells were rinsed twice with PBS and cultured in normal medium with10% FBS for another 20 h [26]. Cell viability was then determined by MTS assay according to the manufacturer’s instruction.

### Protein carbonyl assay

Levels of oxidized proteins in cells were measured by levels of protein carbonyls after reacting with DNPH [27]. Cells were lysed in 200 µl 50 mM Tris-HCl buffer (pH 7.6) containing 5 mM EDTA, 1% NP-40, 0.1% SDS, 20 mM N-ethylmaleimide, and 2 mM 4-(2-Aminoethyl) benzenesulfonyl fluoride hydrochloride. Protein concentrations in the lysates were measured by the bicinchoninic acid method. Equal amounts of cell lysates were incubated with 5 mM DNPH in 1 mM HCl for 15 min at room temperature in the dark and then neutralized with 2 M Tris. After mixing with equal volume of 2× SDS–PAGE loading buffer and boiling for 3 min, equal amounts of total proteins (5 µg/lane) were resolved on 12% SDS–PAGE and transferred to nitrocellulose membranes. The membrane was probed with rabbit antibody against DNPH or mouse monoclonal antibody against β-actin. After incubation with the corresponding horseradish peroxidase-conjugated secondary antibodies, the specific bound antibody was visualized using Super Signal chemiluminescent detection kit (Thermo, Rockford, IL).

### Lipid peroxidation assay

Lipid peroxidation was assessed by measuring the contents of malondialdehyde (MDA) in the cells using an HPLC based method [28]. Cell pellets were homogenized in ice-cold phosphate buffered saline (PBS) containing 0.1% butylated hydroxytoluene (BHT) by sonication using a Branson Sonifier 450 ultrasonicator (Branson, Danbury, CT). Cell lysates were first incubated with NaOH at 60 °C for 30 min, and followed by protein precipitation with trichloroacetic acid and centrifugation. The supernatant was reacted with thiobarbituric acid (TBA) at 95 °C for 1 h. The MDA-TBA adducts were analyzed using an HPLC system equipped with a Varian microsorb100–5 C18 column (150×4.6 mm) and a fluorescence detector set at Ex 515 nm and Em 553 nm. The HPLC mobile phase was phosphate buffer/methanol (13:7, v/v) and the flow rate was set at 0.8 ml/min.

### Analysis of glutathione

Levels of reduced glutathione and oxidized glutathione (GSSG) in the cells were determined by HPLC-ECD method developed by Harvey et al. [29] Potentials were applied from 60 to 960 mV with 60 mV increment. Cell suspensions were mixed with equal volume of 1 M perchloric acid and the cells were lysed by sonication. After centrifugation, the supernatant was diluted in mobile phase A and applied onto HPLC. The concentrations of GSH and GSSG in HLEC were calculated using calibration curves of authentic GSH and GSSG (*R^2^* >0.995). Each sample was analyzed in duplicates. Absolute quantities of authentic GSH and GSSG on the column ranged from 5 to 250 and 0.5 to 50 pmol, respectively.

### Single-cell gel electrophoresis analysis (comet assay)

DNA damage in HLEC was measured using alkaline single-cell gel electrophoresis (Comet assay) as described previously [30-32], with some modifications. Dispersed HLEC were added into 0.5% low melting point agarose and the mixture was layered onto slides precoated with 1.5% normal agarose. Then the slides were immersed in a lysis solution (2.5 mM NaCl, 100 mM EDTA, 10 mM Tris PH 10, 1% N-lauroyl sarcosine sodium, 1% Triton X-100, and 10% DMSO) for 24 h. After alkaline electrophoresis (pH>13), neutralization, and SYBRgreen (Trevigen, Gaithersburg, MD) staining, the slides were examined using a fluorescent microscope at 400× magnification [33]. Every step was performed under indirect light. Slides were coded and analyzed without knowledge of the identity of the sample. DNA damage was measured using computer scoring system (Comet assay IV Software; Perceptive Instruments, Haverhill, Suffolk, UK). To avoid any selection bias, at least 100 cells from each sample were counted and amounts of DNA in the tail and main body were measured by fluorescence intensity. The percentage of DNA in the tail was used to express the amount of DNA damage.

### Statistical analysis

Each experiment was done in triplicate and repeated at least twice. All reported values are expressed as mean±SD. Statistical analyses were performed by using Student’s *t*-test assuming equal variances for all data points (comparison of two groups) or by one-way ANOVA (comparison of multiple groups). A p<0.05 was considered statistically significant.

## Results

### Supplementation of lutein or zeaxanthin have no cytotoxicity to human lens epithelial cells

**Figure 1:**
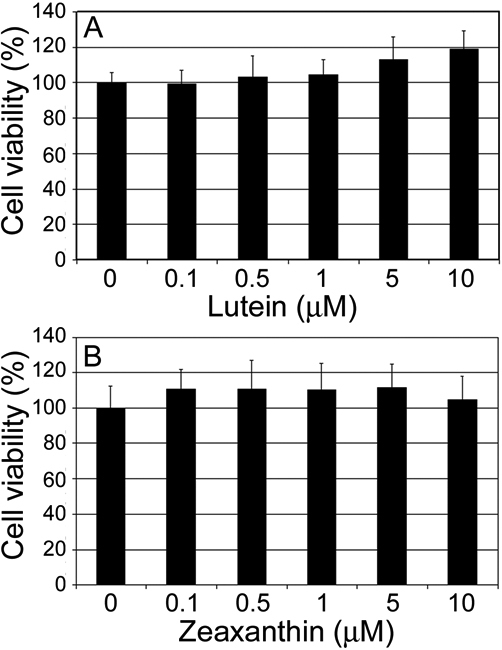
Effects of lutein or zeaxanthin supplementation on cell viability of lens epithelial cells. Subconfluent human lens epithelial cells were incubated with 0, 0.1, 0.5, 1, 5, and 10 µM lutein or zeaxanthin for 48 h and then cell viability was determined. **A**: Effects of lutein supplementation of cell viability. **B:** Effects of zeaxanthin supplementation on cell viability.

Lutein and zeaxanthin are lipid soluble antioxidant nutrients, but their roles in the lens have not been extensively studied. To confirm the safety of lutein and zeaxanthin supplementation, we first determined the effects of lutein and zeaxanthin supplementation on viability of HLEC. We chose to use 0.1 to 10 μM lutein or zeaxanthin in this study according to concentrations of lutein in human plasma (0.1 to 1 μM). In contrast to the data in a previous report [34], we found that supplementation of lutein or zeaxanthin to HLEC had no adverse effects, at least at the range of concentrations tested (Figure 1). Instead, it appeared that supplementation of lutein or zeaxanthin slightly enhanced the cell viability. But the increase in cell viability was not statistically significant. The discrepancy between these results and the previous report may be related to difference in cell types or difference in quality of lutein preparations. It is plausible that the cytotoxicity of lutein reported in the previous report was due to impurities in their lutein preparation.

### Dose-dependent effects of lutein in protecting HLEC from oxidative damage

**Figure 2:**
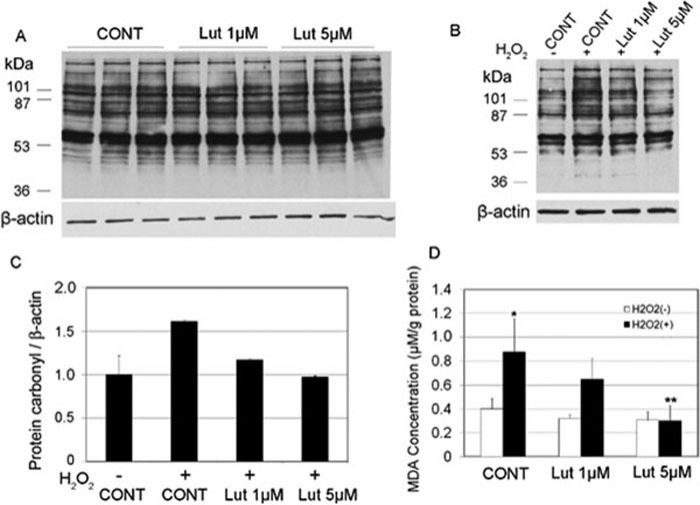
Dose-dependent effects of lutein supplementation on protein and lipid oxidations. Subconfluent human lens epithelial cells were incubated with 0, 1, or 5 µM lutein for 48 h to allow the cells to accumulate lutein. The cells were then treated with or without 100 µM H_2_O_2_ for 1 h. Levels of protein carbonyl and MDA in the cells were determined as described in the “Methods.” **A**: The effects of lutein supplementation on protein carbonyl in HLEC that were not exposed to H_2_O_2_. **B**: The effects of lutein supplementation on protein carbonyl in HLEC that were exposed to 100 µM H_2_O_2_ for 1 h. **C**: Densitometry quantification of western-blotting results in **B** (n=3). **D**: The effects of lutein supplementation on MDA levels (n=6), *Indicates a p<0.05 when comparing H_2_O_2_-exposed groups to the control group that were not treated with H_2_O_2_ and **indicates a p<0.05 when comparing lutein supplemented groups to the unsupplemented group upon exposure to 100 µM H_2_O_2_ for 1h.

To identify a useful dose for lutein supplementation, we first compared the effects of two different concentrations (1 µM and 5 µM) of lutein on protecting HLEC against oxidative damage. Levels of protein carbonyls and lipid peroxidation were used as markers of oxidative damage. In the absence of H_2_O_2_-treatment, the cells contained low levels of protein carbonyls. Supplementation with 1 µM or 5 µM lutein did not alter the protein carbonyl levels in the unstressed cells (Figure 2A). To determine the effects of lutein supplementation against oxidative stress, we determined the effects of lutein supplementation on H_2_O_2_-induced increase in protein and lipid oxidation. Exposure of HLEC to as low as 100 μM H_2_O_2_ induced reproducible increase in levels of protein carbonyls (Figure 2B, Compare lane 2 with lane 1). This level of H_2_O_2_ was detected in the aqueous humor or cataract lenses [35-37] and considered physiologically relevant. Lutein supplementation before oxidative stress prevented the H_2_O_2_-induced increase in protein carbonyls (Figure 2B, Compare lanes 3 and 4 with lane 2). It appears that the protective effects of lutein is dose-dependent as 5 µM lutein provided stronger protections than 1 µM lutein (Figure 2B,C). We next determined the dose-dependent effects of lutein supplementation on lipid peroxidation. In cells that were not exposed to H_2_O_2_, levels of MDA, a lipid peroxidation product, were very low (Figure 2D). Lutein supplementation had little effect on levels of MDA in the absence of oxidative stress (Figure 2D). In contrast, exposure of cells to 100 μM H_2_O_2_ increased the levels of MDA by more than twofold (Figure 2D). Lutein supplementation also suppressed the H_2_O_2_-induced increase in levels of MDA and the protective effects of lutein against lipid peroxidation were dose-dependent, with 5 μM lutein almost completely blocking the H_2_O_2_-induced increase in levels of MDA (Figure 1D). We expected that higher concentrations of lutein would provide stronger protection. However, due to its poor solubility, lutein at concentrations higher than 5 μM in the medium results in precipitation. So we chose to use 5 µM in all the proceeding experiments to compare the effects of lutein, zeaxanthin, and α-tocopherol against H_2_O_2_-induced oxidative damage.

### Lutein, zeaxanthin, and α-tocopherol supplementations prevent H_2_O_2_-induced accumulation of protein carbonyls

**Figure 3:**
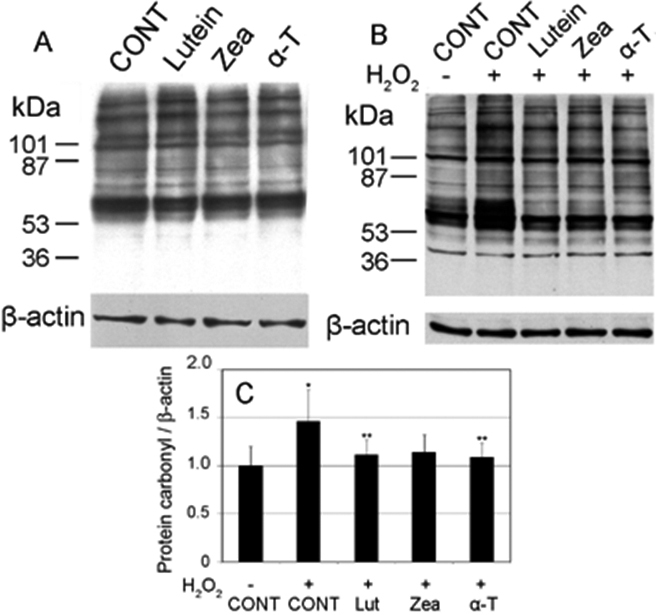
Supplementation with lutein, zeaxanthin, or α-tocopherol prevented H_2_O_2_-induced increase in levels of protein carbonyls in human lens epithelial cells (HLEC). Subconfluent HLEC were pre-incubated with or without 5 µM lutein, zeaxanthin, or α-tocopherol for 48 h and then exposed to 100 µM H_2_O_2_ for 1 h. Levels of protein carbonyls were determined by western blotting after derivatization with DNPH. β-Actin was used as the loading control. **A**: Effects of lutein, zeaxanthin, and α-tocopherol supplementation on levels of protein carbonyls in cells that were not exposed to H_2_O_2_. **B**: Effects of lutein, zeaxanthin, and α-tocopherol supplementation on levels of protein carbonyls in cells that were exposed to 100 µM H_2_O_2_, for 1 h. **C**: Densitometry quantification of western-blotting results in **B** (n=3). *Indicates a p<0.05 when comparing H_2_O_2_-exposed groups to the control group that were not treated with H_2_O_2_ and **indicates a p<0.05 when comparing lutein, zeaxanthin or α-tocopherol supplemented groups to the unsupplemented group upon exposure to 100 µM H_2_O_2_ for 1h.

To evaluate the antioxidant capacities of lutein and zeaxanthin, we compared the efficacies of lutein and zeaxanthin against oxidative damage to that of α-tocopherol, a form of vitamin E and a well studied lipid antioxidant. As shown in Figure 3A, supplementation of HLEC with 5 μM lutein, zeaxanthin or α-tocopherol for 48 h had little effects on levels of protein carbonyls in cells that were not exposed to H_2_O_2_. However, supplementation of the same levels of lutein, zeaxanthin, or α-tocopherol substantially prevented the H_2_O_2_-induced increase in levels of protein carbonyls (Figure 3B). The efficacies of lutein, zeaxanthin, and α-tocopherol in preventing H_2_O_2_-induced accumulation of protein carbonyls were comparable (Figure 3B,C).

### Lutein, zeaxanthin and α-tocopherol supplementation decreases H_2_O_2_-induced lipid peroxidation

**Figure 4:**
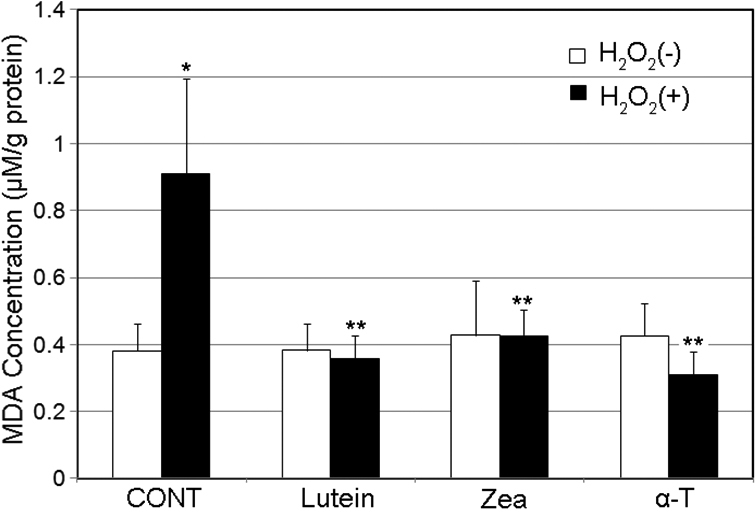
Supplementation with lutein, zeaxanthin, or α-tocopherol suppressed H_2_O_2_-induced lipid peroxidation in human lens epithelial cells (HLEC). Subconfluent HLEC were pre-incubated with or without 5 µM of lutein, zeaxanthin or α-tocopherol for 48 h and then exposed to 100 µM H_2_O_2_ for 1 h. MDA levels were determined by an HPLC system. Each value represents the mean±SD (n=6). *Indicates a p<0.05 when compared to cells not treated H_2_O_2_ and **indicates a p<0.05 when comparing to the group that were not supplemented with any of these nutrients and treated with the same level of H_2_O_2_.

As shown in Figure 4, in the absence of oxidative insult, supplementation of lutein, zeaxanthin, or α-tocopherol for 48 h did not alter the basal levels of MDA in HLEC. H_2_O_2_ exposure significantly increased concentrations of MDA in the control group. In contrast, supplementation of HLEC with 5 µM lutein, zeaxanthin, or α-tocopherol for 48 h before H_2_O_2_ exposure completely prevented the H_2_O_2_-induced increase in levels of MDA. The protective effects of lutein, zeaxanthin, and α-tocopherol on H_2_O_2_-induced lipid peroxidation were also comparable.

### Lutein, zeaxanthin, and α-tocopherol prevent H_2_O_2_-induced DNA damage

**Figure 5:**
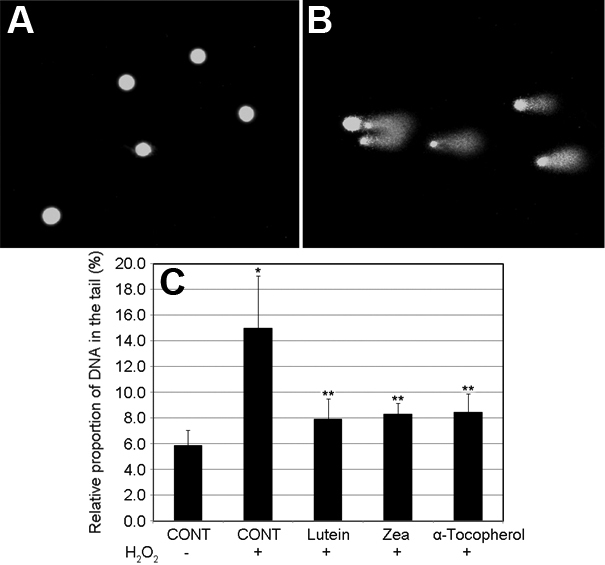
Supplementation with lutein, zeaxanthin or α-tocopherol suppressed H_2_O_2_-induced DNA damage in human lens epithelial cells (HLEC). Subconfluent HLEC were incubated with or without 5 µM of lutein, zeaxanthin or α-tocopherol for 48 h and then exposed to 100 µM H_2_O_2_ for 1 h. **A**: Fluorescence micrograph of nuclei of normal HLEC. **B**: Fluorescence micrograph of nuclei of oxidative insulted HLEC. **C**: Quantification of DNA damage. Each value represents the mean±SD (n=6). *Indicates a p<0.01 when comparing with cells not treated with H_2_O_2_ and **indicates a p<0.05 comparing with unsupplemented cells that were exposed to the same level of H_2_O_2_.

To comprehensively investigate the protective effects of lutein and zeaxanthin against oxidative damage, we further determined the effects of lutein, zeaxanthin, and α-tocopherol on DNA damage using the comet assay. Figure 5A,B show the fluorescence micrographs of normal HLEC nuclei and H_2_O_2_- damaged HLEC nuclei with comet tails. In the absence of oxidative stress, most of the cell nuclei appeared intact (Figure 5A). After exposure to H_2_O_2_, a substantial amount of the nuclear DNA migrated as comet tails, indicating broken DNA strains (Figure 5B). The effects of lutein, zeaxanthin, and α-tocopherol supplementation on oxidation-induced DNA damage were determined by calculating the proportion of DNA in the comet tails (Figure 5C). In unsupplemented cells, H_2_O_2_ exposure increased the proportion of DNA in the tails as compared to the group not exposed to oxidative stress (Figure 5C). Supplementation of the cells with 5 µM lutein, zeaxanthin, or α-tocopherol for 48 h before oxidative stress significantly prevented H_2_O_2_-induced increase in levels of damaged DNA (Figure 5C). The protective effects of lutein and zeaxanthin were similar to that of α-tocopherol.

### Supplementation with lutein, zeaxanthin, or α-tocopherol increases levels of intracellular GSH in HLEC

To investigate effects of lutein, zeaxanthin, or α-tocopherol supplementation on intracellular redox status, we compared the levels of GSH and the GSH:GSSG ratio in unstressed cells and oxidative insulted cells. In the absence of oxidative stress, supplementation with α-tocopherol, but not with lutein or zeaxanthin, slightly increased intracellular levels of GSH and GSSG (Table 1). The intracellular redox status (GSH:GSSG ratios) were lower in the supplemented groups as compared to the controls due to the increase in levels of GSSG in supplemented cells. In contrary to our expectation, we found that exposure to 100 μM H_2_O_2_ for 1 h increased levels of GSH and GSSG slightly (Table 1). Supplementation of lutein, zeaxanthin, or α-tocopherol also slightly increased levels of GSH in H_2_O_2_-exposed cells. The effects of zeaxanthin and α-tocopherol supplementation were stronger than lutein supplementation in this aspect (Table 1). In contrast with the depression of GSH/GSSG ratios noted in unstressed assays, supplementation with these nutrients also increased GSH:GSSG ratios in H_2_O_2_-challenged cells (Table 1). These data suggest that supplementation of lutein, zeaxanthin, or α-tocopherol improves intracellular redox status, particularly under oxidative stress. The supplementation-related increase in GSH:GSSG ratios in oxidatively stressed cells were mainly due to increase in GSH levels.

**Table 1:** Effects of lutein, zeaxanthin and α-tocopherol supplementations on GSH and GSSG levels in human lens epithelial cells (HLEC).

**H_2_O_2_ (µM)**	**Nutrients (5 µM)**	**GSH (µmol/g protein)**	**GSSG (µmol/g protein)**	**GSH/GSSG**
0	Control	9.83±2.16	0.65±0.30	15.0
	Lutein	9.33±1.77	0.73±0.25	12.7
	Zeaxanthin	11.11±2.19	0.89±0.22	12.5
	α-tocopherol	12.85±1.25*	1.03±0.08*	12.5
100	Control	11.95±1.32	0.92±0.30	13.0
	Lutein	13.88±2.07	0.92±0.29	15.0
	Zeaxanthin	18.11±3.65*	1.12±0.30	16.2
	α-tocopherol	17.83±3.85*	0.94±0.21	18.9

HLEC were supplemented with or without 5 µM of lutein, zeaxanthin or α-tocopherol for 48 h and then exposed to 100 µM H_2_O_2_ for 1 h. Levels of GSH and GSSG were determined by HPLC. Each value is expressed as mean±SD (n=6), *Indicates a p<0.05 when compared with control groups that were treated with the same level of H_2_O_2_.

### Lutein and zeaxanthin supplementation cannot ameliorate H_2_O_2_-induced cytotoxicity

**Figure 6:**
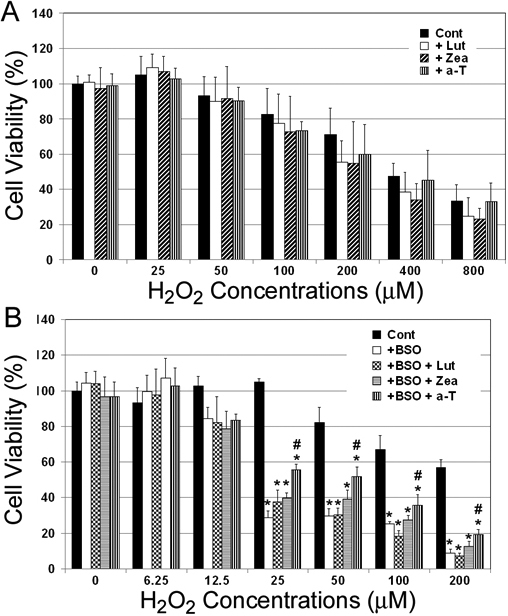
The effects of lutein, zeaxanthin, or α-tocopherol supplementation on cell viability upon exposure to H_2_O_2_. **A**: Subconfluent human lens epithelial cells (HLEC) were incubated with or without 5 µM of lutein, zeaxanthin or α-tocopherol (α-T) for 48 h and then exposed to indicated concentrations of H_2_O_2_ for 1 h. **B**: HLEC were cultured with 1 mM D-L(R:S) buthionine sulfoximine (BSO) in the presence of 5 μM lutein, zeaxanthin or α-tocopherol for 48 h. The cells were exposed to the indicated concentrations of H_2_O_2_ for 1 h in a BSO- and nutrients-free medium. Cell viabilities were determined MTS assay after 20 h recovery in normal medium (n=6). *Indicates that p<0.05 when compared with control group that were not treated with BSO and # indicate a p<0.05 when comparing with the group that was treated with BSO, but not supplemented with any of these nutrients when exposed to the same levels of H_2_O_2_.

The above data showed that supplementation of HLEC with lutein or zeaxanthin reduced oxidative damage to protein, lipids and DNA. To further investigate the protective effects of lutein and zeaxanthin supplementation, we tested their effects on H_2_O_2_-induced cytotoxicity to HLEC. To do so, HLEC were preincubated with 5 µM of lutein, zeaxanthin or α-tocopherol for 48 h and then exposed to the indicated concentrations of H_2_O_2_ in serum-free medium for 1 h. Cell viability was determined by MTS assay. MTS assay measures the activities of mitochondrial reductases that are proportional to the number of viable cells. Because these enzymes can be reversibly inhibited by H_2_O_2_, we measured the cell viability after 20 h recovery in normal medium. As shown in Figure 6A, supplementation of lutein or zeaxanthin before H_2_O_2_ exposure did not prevent H_2_O_2_-induced loss of cell viability. In fact, it appeared that lutein and zeaxanthin supplementation slightly enhanced the toxicity of H_2_O_2_. But this apparent difference in cytotoxicity was not statistically significant. Consistent with our previous findings in rabbit lens epithelial cells [26], supplementation of the cells with α-tocopherol also failed to protect the cells from H_2_O_2_-induced loss of cell viability (Figure 6A).

### Supplementation with lutein or zeaxanthin cannot restore the resistance of GSH depleted cells to H_2_O_2_-induced loss of cell viability

We previously showed that in rabbit lens epithelial cells GSH depletion dramatically increased susceptibility to H_2_O_2_-induced loss of cell viability and that supplementation of ascorbate and α-tocopherol can restore the resistance of GSH-depleted cells to oxidative stress [26]. Here we determined the effects of lutein or zeaxanthin supplementation on susceptibility of GSH-depleted HLEC to oxidative stress using cell viability as an indicator. Intracellular GSH was depleted by treatment of the cells with BSO to block GSH biosynthesis. The effects of BSO on cellular levels of GSH were dose-dependent. Treatment of HLEC with 1 mM BSO for 48 h resulted in >60% reduction of cellular glutathione. As shown in Figure 6B, treatment of HLEC with BSO alone had little effect on cell viability under these conditions. However, BSO treatment rendered the cells more sensitive to oxidative stress. HLEC not treated with BSO withstood 50 μM H_2_O_2_ for 1 h without significant loss of cell viability (Figure 6B). Treatment of GSH replete cells with 200 μM H_2_O_2_ for 1 h only resulted in ~40% reduction of cell viability of control cells (Figure 6). In contrast, treatment of GSH-depleted cells with as low as 25 μM H_2_O_2_ for 1 h resulted in ~70% decline in cell viability (Figure 6B). Exposure of GSH-depleted cells to 200 μM H_2_O_2_ resulted in as much as 90% reduction in cell viability. To determine the effects of lutein or zeaxanthin supplementation on sensitivity of GSH-depleted cells to exogenous oxidative stress, HLEC were incubated with 1 mM BSO and 5 μM lutein, zeaxanthin or α-tocopherol for 48 h and then the cells were exposed to the indicated concentrations of H_2_O_2_ in serum-free medium for 1 h. Cell viability was determined after 20 h recovery. As shown in Figure 6B, supplementation with lutein or zeaxanthin did not alter the susceptibility of GSH-depleted cells to H_2_O_2_-induced cell death. In contrast, supplementation with the same level of α-tocopherol partially restored the resistance of GSH-depleted HLEC to H_2_O_2_-induced loss of cell viability. For example, supplementation with α-tocopherol to GSH-depleted cells increased cell viability from 30% to 55% upon exposure to 25 μM H_2_O_2_. The data indicate that supplementation with α-tocopherol, but not lutein or zeaxanthin, partially compensates for GSH-depletion in response to oxidative stress.

## Discussion

Oxidative stress is the result of an imbalance in pro-oxidant and antioxidant homeostasis that arises from a persistent elevation of reactive oxygen species production or a decline in capacities of antioxidant defenses. Oxidative stress plays an important role in cataractogenesis and H_2_O_2_ is one of major oxidants that appears to contribute to cataract formation [12,13,38-41]. Lens epithelial cells are the primary targets of oxidants in the aqueous humor because the aqueous is the proximate fluid that contacts the epithelial surface of the lens [42]. Exposure of lens epithelial cells to oxidants, such as H_2_O_2_, results in protein oxidation, lipid peroxidation, DNA damage and cell death [39-41]. In theory, protection of lens cells, particularly epithelial cells, from oxidative damage is a valid strategy for cataract prevention. Results from this work indicate that supplementation of lens epithelial cells with lutein or zeaxanthin can effectively block H_2_O_2_-induced protein oxidation, lipid peroxidation and DNA damage (Figure 2, Figure 3, Figure 4, and Figure 5). The protective effects of lutein or zeaxanthin were comparable to that of the same levels of α-tocopherol.

Both lutein and zeaxanthin are found in the human lens. The concentrations of lutein/zeaxanthin in human lenses were reported 0.03–0.07 μM [10,43]. In the lens, lutein and zeaxanthin are not evenly distributed. The epithelial layer contains the highest concentration of lutein/zeaxanthin and the nuclear core contains the lowest concentration of these nutrients [11]. Given the antioxidant property of lutein and zeaxanthin [44], an increase in levels of lutein and zeaxanthin in lens will increase the antioxidant capacities and reduce oxidative damage. The enhancement of antioxidant capacity in the lens via an elevation of lutein and zeaxanthin concentrations in the lens may contribute to the reduced risk of cataract in individuals with higher dietary lutein or zeaxanthin intake [3,4,6-8]. Since the only source of lutein and zeaxanthin in the body is dietary intake, it is reasonable to hypothesize that the concentrations of these nutrients in the lens and other tissues are related to status of long-term dietary intake. It has been reported that macular pigment optical density (which noninvasively measures the concentrations of lutein/zeaxanthin in the retina) increases in response to long-term dietary supplementation [45]. Our unpublished data also indicate that levels of lutein, zeaxanthin and α-tocopherol in lenses of donor eyes were positively correlated to their concentrations in the retinas. However, the direct evidence for relationship between dietary intake and lens concentrations of these nutrients is still not available. The mechanisms that govern transportation of lutein and zeaxanthin to the lens also remain to be elucidated. We speculate that these compounds are transported to the lens through aqueous humor. These nutrients may also enter into lens via vitreous body, as retina contains the highest concentration of lutein/zeaxanthin in the body. In plasma, these lipophilic nutrients are mainly carried by lipoproteins. Aqueous humor also contains lipoproteins, which may play an important role in transporting lipophilic nutrients to the lens. Future studies are warranted to establish the relationship between dietary intake and lens concentrations of these nutrients and to elucidate the mechanisms by which these nutrients are transported to the lens.

In addition to quenching reactive oxygen species directly, lutein, zeaxanthin and α-tocopherol may prevent protein, lipid or DNA from oxidative damage by regulating other cellular antioxidant systems. Glutathione is one of the major intracellular antioxidants in the lens and plays an important role in protecting cells from oxidative damage [46-51]. Our data showed that supplementation of lutein, zeaxanthin and α-tocopherol to HLEC increased the levels of GSH and GSSG, particularly after H_2_O_2_ challenge (Table 1). Although the extent of the increase in glutathione was not dramatic, it was observed constantly. Similar effects of α-tocopherol supplementation on intracellular glutathione were also observed in HaCaT keratinocytes [52]. Furthermore, a previous publication reported that oral supplementation of vitamin E to human or rabbit increased levels of total glutathione in red blood cells and in the lens [53]. It remains unclear how lutein, zeaxanthin or α-tocopherol in μM levels alters concentrations of glutathione that is in mM levels. One of the mechanisms is that that lutein, zeaxanthin or α-tocopherol directly or indirectly regulates glutathione synthesis and therefore glutathione levels. It was reported that α-tocopherol treatment increased mRNA levels of gamma-glutamylcysteine synthetase, the rate-limiting enzyme for glutathione biosynthesis. Lutein and zeaxanthin may alter glutathione levels via a similar mechanism as α-tocopherol, but this needs to be determined in future studies.

Although supplementation of lutein, zeaxanthin, or α-tocopherol effectively prevented H_2_O_2_-induced oxidation of protein, lipid and DNA, supplementation of these nutrients did not block H_2_O_2_-induced loss of cell viability (Figure 6A). This clearly indicates that some type of damage that triggers cell death was not prevented by these antioxidant nutrients. In addition to protein, lipid and DNA damage, disturbance of the cellular signaling systems or mitochondrial functions could also trigger cell death, particularly through apoptosis. It is known that mitochondria are susceptible targets of oxidative stress and damage to mitochondria results in release of cytochrome C and apoptosis. It is plausible that supplementation of these levels of lutein, zeaxanthin or α-tocopherol is not sufficient to protect these sensitive targets from oxidative damage and thus H_2_O_2_-induced loss of cell viability. Future studies on the effects of lutein or zeaxanthin supplementation on H_2_O_2_-induced alterations in cell signaling pathways or mitochondrial functions will help explain why supplementation of these antioxidant nutrients blocks H_2_O_2_-induced oxidation of protein, lipid and DNA, but not blocks H_2_O_2_-induced loss of cell viability.

We also determined the ability of lutein and zeaxanthin supplementation on compensation for GSH depletion in this study. Consistent with our previous work [26], depletion of intracellular GSH in HLEC by treatment with BSO greatly increased the susceptibility to H_2_O_2_-induced cell death. Supplementation with α-tocopherol partially restored the resistance of GSH depleted cells. However, supplementation with lutein or zeaxanthin could not restore the resistance of GSH-depleted HLEC to oxidative stress. These data suggest that although lutein and zeaxanthin have comparable capacities to that of α-tocopherol in protecting protein, lipid and DNA from oxidative damage, they cannot replace α-tocopherol in compensating for GSH depletion. It appears that the interactions between α-tocopherol and GSH are unique. α-Tocopherol may spare the residual GSH in the GSH-depleted cells or α-tocopherol and GSH have common targets of action within cells and these targets are not shared by lutein or zeaxanthin. The details of these interactions and the targets of protection remain to be elucidated. Better understanding of the networks of intracellular antioxidant defense system would help to develop strategies to prevent oxidative damage and associated diseases, such as cataract.
